# A Practical Case of Monitoring Older Adults Using mmWave Radar and UWB

**DOI:** 10.3390/s26020681

**Published:** 2026-01-20

**Authors:** Gabriel García-Gutiérrez, Elena Aparicio-Esteve, Jesús Ureña, José Manuel Villadangos-Carrizo, Ana Jiménez-Martín, Juan Jesús García-Domínguez

**Affiliations:** Department of Electronics, University of Alcalá, 28805 Alcalá de Henares, Spain; elena.aparicio@uah.es (E.A.-E.); jm.villadangos@uah.es (J.M.V.-C.); ana.jimenez@uah.es (A.J.-M.); jjesus.garcia@uah.es (J.J.G.-D.)

**Keywords:** Ultra-Wideband (UWB), millimeter-wave (mmWave) radar, indoor localization, elderly monitoring, partially constrained Kalman filter, short-term average/long-term average (STA/LTA)

## Abstract

Population aging is driving the need for unobtrusive, continuous monitoring solutions in residential care environments. Radio-frequency (RF)-based technologies such as Ultra-Wideband (UWB) and millimeter-wave (mmWave) radar are particularly attractive for providing detailed information on presence and movement while preserving privacy. Building on a UWB–mmWave localization system deployed in a senior living residence, this paper focuses on the data-processing methodology for extracting quantitative mobility indicators from long-term indoor monitoring data. The system combines a device-free mmWave radar setup in bedrooms and bathrooms with a tag-based UWB positioning system in common areas. For mmWave data, an adaptive short-term average/long-term average (STA/LTA) detector operating on an aggregated, normalized radar energy signal is used to classify micro- and macromovements into bedroom occupancy and non-sedentary activity episodes. For UWB data, a partially constrained Kalman filter with a nearly constant velocity dynamics model and floor-plan information yields smoothed trajectories, from which daily gait- and mobility-related metrics are derived. The approach is illustrated using one-day samples from three users as a proof of concept. The proposed methodology provides individualized indicators of bedroom occupancy, sedentary behavior, and mobility in shared spaces, supporting the feasibility of combined UWB and mmWave radar sensing for longitudinal routine analysis in real-world elderly care environments.

## 1. Introduction

According to recent Eurostat data, people aged 65 years and over already account for about one-fifth of the European Union population (21.1% in 2022), compared with roughly 18% ten years earlier [1]. The share of people aged 80 years and over also continues to grow and is projected to more than double over the course of the century, from around 6% in 2022 to over 15% by 2100 [1]. This demographic shift places growing pressure on health and long-term care systems and reinforces the need for technological solutions that enable safe and healthy aging, both at home and in residential settings, where many older adults prefer to age in place.

In this context, a scoping review of in-home monitoring technologies highlights how smart-home systems are being used to track daily activities, detect abnormal behaviors, follow cognitive decline, identify falls, estimate indoor position, and assess sleep quality in older adults [2]. These systems typically combine wearable devices, environmental sensors, and home automation to provide objective information on functional, cognitive, and social status. In addition, recent surveys underline the relevance of non-contact and multimodal approaches; [3] reviews indoor human monitoring systems that integrate cameras, WiFi, Ultra-Wideband (UWB), and radar with a specific focus on elderly care, and emphasizes that, although there is a growing trend in non-contact sensing, real-world, long-duration deployments in residential environments remain relatively scarce.

From this perspective, several multisensory platforms have been proposed to jointly measure activity and location. A long-term monitoring system that fuses inertial measurements with WiFi- and ultrasound-based localization to derive daily physical activity levels and routines of older adults in different living environments is presented in ref. [4]. A wireless sensor network for people with mild cognitive impairment that integrates indoor and outdoor localization with step detection, identifies anomalous changes in daily routines, and provides caregivers with alerts in risky situations is described in ref. [5]. These works illustrate the potential of combining activity metrics with spatial information to detect early physical or cognitive deterioration.

Radio-frequency (RF)-based solutions have emerged as a promising alternative for indoor monitoring because they can provide detailed information on presence and movement while limiting the collection of visually identifiable data. In this work, “privacy” refers to the use of non-visual RF measurements (e.g., radar returns and tag positions) that are processed into low-level presence/motion/position indicators, rather than capturing images or other directly identifiable content. In this regard, UWB-based systems, both in the form of tag-based real-time location systems (RTLS) and monostatic radars, have been applied to indoor activity monitoring in elderly care, enabling the estimation of trajectories and activity levels with high spatial resolution [6,7]. BLE–UWB hybrid systems for behavioral analysis of community-dwelling older adults show that combining coarse and fine localization can optimize accuracy, power consumption, and cost [8]. At the same time, UWB radar surveys highlight the ability of these devices to detect subtle vital-sign-related micro-movements such as breathing, as well as gross movements, through walls and in low-light conditions [9]. Millimeter-wave (mmWave) frequency-modulated continuous-wave (FMCW) radars have also been proposed for human activity monitoring and telemedicine, demonstrating their capacity to capture posture changes and movement patterns in a fully device-free manner [10].

Recent works have investigated the joint use of UWB and frequency-modulated continuous-wave (FMCW)/millimeter-wave (mmWave) radar to improve indoor localization robustness under challenging propagation conditions (LOS/NLOS), typically through Extended Kalman Filter (EKF)-based fusion and short controlled trajectories [11,12]. While these studies mainly focus on instantaneous positioning accuracy, the present work targets long-term residential monitoring and the extraction of occupancy and mobility indicators by combining device-free mmWave sensing in private rooms (bedroom/bathroom) with tag-based UWB trajectories in shared areas.

Despite these advances, many studies still focus on a single sensing modality (for example, UWB localization or mmWave radar independently) or evaluate multimodal fusion only in short, controlled trials; consequently, they often rely on small samples and are restricted to brief experimental campaigns in laboratory or pilot apartments [2,3,6]. Although several multisensory platforms combining activity and location sensing have been deployed in real homes and residential facilities [4,5], there are still very few examples of systems that integrate UWB localization in common areas with mmWave radar-based presence and activity monitoring in private rooms over extended periods in real senior residences.

### Indoor Monitoring Technologies

Indoor monitoring technologies for supporting older adults cover a broad spectrum of solutions. A scoping review of in-home monitoring technologies identifies six main functions in smart-home systems for aging in place: monitoring daily activities, detecting abnormal behaviors, screening for cognitive impairment, detecting falls, providing indoor positioning, and assessing sleep quality [2]. These functions are implemented through combinations of wearable devices, passive infrared and contact sensors, smart meters, and other home automation components. A recent survey on non-contact multimodal monitoring shows how cameras and radio devices (WiFi, UWB, mmWave radar) can be combined to improve robustness and accuracy in elderly monitoring [3].

Wearable-based approaches offer direct measurement of physical activity and physiological parameters but depend on user adherence and can be perceived as intrusive, especially by frail individuals or those with cognitive impairment [2]. Environmental sensors such as PIR detectors, door contacts, or energy meters are less obtrusive and easier to integrate into existing homes, but they only provide indirect and low-resolution information about activity. RF localization systems exploit the interaction between the human body and electromagnetic waves to infer position, presence, and movement without cameras, which can facilitate acceptance and compliance with privacy requirements [3,9].

Within these non-contact technologies, UWB systems have been used both as tag-based RTLS to track older adults in homes and care facilities [7,8] and as monostatic radars to estimate activity and presence in instrumented apartments [6,9]. mmWave frequency-modulated continuous-wave (FMCW) radars can detect macro- and micro-movements at room scale and have been proposed for activity monitoring and telemedicine applications [10]. Recent multisensory systems combine this information with inertial, WiFi, or ultrasonic sensing to characterize activity patterns and routines of older adults living in the community [4,5].

The present paper reports a practical case of monitoring older adults in a senior living residence using a localization system that combines UWB and mmWave radar [13]. Specifically, we focus on the data-processing methodology applied to long-term data collected by this system to estimate quantitative mobility parameters for the monitored residents. On the one hand, a device-free mmWave radar setup is used in bedrooms and bathrooms, where an adaptive STA/LTA detector fed by an aggregated, normalized energy signal is applied to classify radar micro- and macromovements into bedroom occupancy and non-sedentary activity episodes. On the other hand, a UWB tag-based positioning system deployed in common areas is processed with a partially constrained Kalman filter using a nearly constant velocity dynamics model and map information, yielding smoothed trajectories from which daily gait and mobility metrics are derived. Together, these processing pipelines provide individualized indicators of bedroom occupancy, sedentary behavior, and mobility in shared spaces, supporting routine analysis in a real-world residential environment.

The remainder of this paper is structured as follows: Section 2 describes the localization system architecture, comprising the UWB and mmWave devices and the data acquisition layer. Section 3 presents the methodology for mmWave data processing and the obtained results for occupancy and activity parameters. Section 4 describes UWB data processing and the corresponding mobility metrics, and Section 5 concludes this paper by summarizing the findings and outlining directions for future research.

## 2. System Architecture

**Figure 1:**
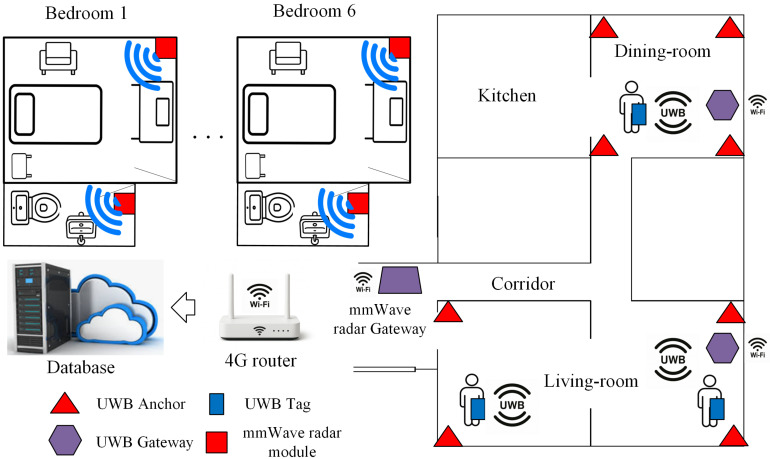
General diagram of the installed localization system [13].

Figure 1 shows the deployment map of the localization system within the senior living residence, including the UWB anchor network in the common areas, the mmWave radar devices installed in private spaces (bedrooms and bathrooms), and the gateway and router nodes that manage data acquisition and connectivity. This layout provides combined coverage of shared and private zones, enabling continuous monitoring of mobility and room-level activity. The following subsections briefly describe the mmWave radar subsystem, the UWB subsystem, and the data acquisition and communication architecture implemented in this work. Further details of the deployed system can be found in ref. [13].

### 2.1. mmWave Radar System

The radar subsystem is based on the commercial off-the-shelf (COTS) RoomSense IQ module, which is built on top of the mmWave radar sensor HLK-LD2410 [14,15]. This device has been installed in the private areas of the senior living residence, namely each user’s bedroom and bathroom, to enable non-intrusive, device-free monitoring. Each module provides a binary presence signal and radar energy levels across multiple distance gates, which are later used to classify micro- and macromovements and to derive bedroom occupancy, bathroom use, and activity-related indicators. Radar data are managed by a dedicated gateway node, which is responsible for local acquisition, temporary storage, and secure forwarding to the remote server.

### 2.2. UWB System

The UWB subsystem is based on the Qorvo DWM1001 UWB transceiver and a real-time localization system (RTLS) built on the proprietary Positioning and Networking Stack (PANS 2.0) [16,17]. The system provides tag-based positioning in the common areas of the residence. Each user wears a UWB tag that performs ranging with a set of fixed anchors and reports its three-dimensional position (x,y,z) together with a timestamp to a gateway node running the manufacturer’s RTLS software stack. This setup enables continuous tracking of indoor mobility, including transitions between shared spaces and time spent in different zones. The gateway node aggregates UWB position data and exposes them through standard interfaces for subsequent processing.

### 2.3. Data Acquisition and Gateway Architecture

The mmWave radar and UWB subsystems are interfaced through dedicated gateway nodes deployed in the residence (Figure 1). Each gateway acts as a protocol translator and local data aggregator, enabling continuous sensing while decoupling low-level device management from data storage and subsequent analysis.

For UWB localization, multiple tags (one per user) communicate with an array of fixed anchors. The gateway node bridges the RTLS stack to the local network and retrieves timestamped 3D position estimates (x,y,z) per tag at a nominal sampling period of 1 s. UWB position samples are produced only when the tag is within the coverage area of the anchor network; therefore, time intervals with no UWB output are treated as missing data rather than inactivity. The UWB gateway runs the RTLS services, buffers data locally, and exposes the position data to the local network through application-level services.

mmWave radar units deployed in personal spaces publish presence indicators and energy-related signals at 1 Hz through MQTT topics and WebSocket endpoints. The mmWave gateway collects these data, buffers them locally, and forwards them for centralized storage.

Both gateways transmit buffered data to a remote storage server over a secure VPN tunnel, providing centralized data management and enabling remote access for research and clinical analysis without requiring inbound connectivity to the residence network.

In addition to the functional architecture, practical deployment aspects such as cost and power are relevant in real residences.

### 2.4. Practical Considerations (Cost and Power)

The deployment uses commercial off-the-shelf (COTS) hardware: 12 RoomSense IQ mmWave radar modules in private rooms, a Qorvo DWM1001 UWB RTLS with 8 anchors and 7 wearable tags (one per user), and three Raspberry Pi gateways (one Raspberry Pi 5 and two Raspberry Pi 3) for data acquisition and forwarding. Fixed devices (gateways, anchors, radars) are mains-powered for continuous operation, while the tags are battery-powered and require periodic recharging.

Using current distributor prices and vendor power specifications for the RoomSense IQ radar modules, the DWM1001-DEV UWB boards, and the Raspberry Pi 5, our deployment (12 radars, 8 anchors, 7 tags, and 3 gateways) corresponds to a hardware bill of materials on the order of 1–1.5 kEUR and a continuous mains power budget of only a few tens of watts (roughly 15–35 W), with both cost and power scaling approximately linearly with the number of instrumented rooms and the required UWB anchor density [14,16,18].

Overall, this makes the system practically deployable in multi-room facilities, as additional rooms primarily incur the per-room cost and power of one radar module plus any extra UWB anchors needed to maintain reliable coverage in shared areas.

### 2.5. Database Generation and Synchronized Dataset Structure

UWB and mmWave radar data are consolidated into a synchronized database. The dataset comprises UWB tags for $N_{u}=7$ users (one tag per user in the monitored shared areas), while mmWave radar data are available for $N_{u}=6$ users in their personal spaces. The database covers 86 days of monitoring, as reported in previous work [13].

Both the UWB system and the mmWave radar nodes generate timestamps in Unix epoch time directly from the acquisition host clock. Therefore, no synchronization is needed. To build the synchronized database, all records are converted to a common time base and binned at 1 Hz; if a modality has no sample in that bin, the entry is left as missing.

#### 2.5.1. Signals and Variables

**Figure 2:**
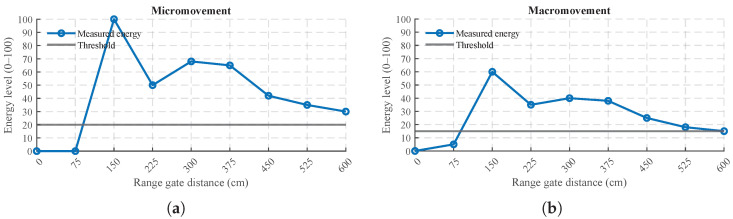
Example of the mmWave module energy output reported in nine fixed 1D range gates along the radar line-of-sight. Each gate aggregates motion energy from targets located within that distance interval, without angular/lateral resolution [14,15]. (**a**) Micromovement energy across the nine 1D range gates (0–6 m at 0.75 m spacing). (**b**) Macromovement energy across the nine 1D range gates (0–6 m at 0.75 m spacing).

The synchronized dataset contains two main groups of variables: (i) UWB localization, including per-tag timestamps and position estimates (x,y,z) (when available); and (ii) mmWave radar monitoring, including a binary presence signal and radar energy signals. The radar energy signal spans a 0–6 m range sampled at nine range gates at 0.75 m spacing. The resulting database therefore provides trajectories in shared living spaces (UWB) and presence states together with energy levels associated with micro- and macro-movements (mmWave) in personal spaces. These range gates correspond to nine fixed 1D distance intervals along the radar line-of-sight (0–6 m in steps of 0.75 m). The module does not provide angular bins or lateral localization; therefore, each gate aggregates motion energy from any target located within that distance interval, irrespective of its azimuth within the antenna field of view, as shown in Figure 2.

#### 2.5.2. Sampling and Missing Data

Both modalities are acquired at a nominal sampling period of 1 s. However, UWB position samples are reported only when the tag is within the anchor coverage area. Consequently, the UWB data can contain temporal gaps due to the user being outside coverage or transient RF/measurement conditions. These intervals are explicitly stored as missing data (no samples) and are not interpreted as sedentary behavior. In contrast, the mmWave gateway provides presence and energy-related signals at 1 Hz whenever the radar units are operational.

#### 2.5.3. Device-to-User Association

A fixed device assignment is maintained throughout this study: for each user we know the corresponding UWB tag identifier and the identifiers of the mmWave radar units installed in that user’s personal spaces (e.g., bedroom and bathroom). This mapping enables subject-specific analysis and facilitates interpreting radar-derived presence in private rooms together with UWB-derived mobility in shared areas.

#### 2.5.4. Temporal Alignment

All records are timestamped at acquisition and converted to a common time base. Data are aligned by their timestamps and, when required, resampled to a fixed 1 Hz grid while preserving missing intervals.

#### 2.5.5. Data Organization and Anonymization

Data are stored remotely in day-wise files and indexed by pseudonymous user/tag identifiers (e.g., User 1–User $N_{u}$). No personally identifying information is included in the research database, and any mapping between hardware IDs and participants is maintained separately under controlled access.

## 3. Processing mmWave Radar Data

As mentioned in previous sections, the mmWave radar provides a binary presence variable (0/1) and radar energy signals scaled from 0 to 100, representing the relative strength of micro-movements (e.g., breathing) and macro-movements (e.g., walking) across nine fixed 1D range gates covering 0–6 m, spaced by 0.75 m, as provided by the module [14,15].

Using these signals, occupancy parameters are derived separately for private areas, namely the bathroom and the bedroom. In the bathroom, temporal patterns of occupancy are obtained from the fused binary presence signal (0/1) produced by combining mmWave radar and PIR data, which is sufficient to count well-delimited visits based on enter/exit events. In contrast, bedroom monitoring requires continuous characterization of micromovements in order to capture activity levels and periods of non-sedentary behavior. The fused binary signal is inherently limited by the inability of PIR sensors to detect stationary occupants, which is particularly critical in older adults, who often exhibit low-amplitude movements and long periods of immobility. Therefore, bedroom-related occupancy parameters rely on radar energy levels from the nine distance gates, which are processed using an adaptive short-term average/long-term average (STA/LTA) detector to identify micro- and macro-movements, reduce dependence on PIR events, and quantify non-sedentary activity.

A detailed description of the processing of the binary presence data and mmWave radar energy signals using the adaptive STA/LTA detector is provided in the following sections.

### 3.1. Data Processing for Binary Presence Data

Presence is used to derive the daily number of bathroom visits, distinguishing between daytime and nighttime events. Nighttime visits are interpreted as a proxy for nocturia episodes in older adults, which have been associated with an increased risk of falls and fractures [19]. In parallel, daytime visit counts can be used to establish an individual baseline of habitual bathroom use for medium/long-term analysis, as deviations from these patterns may signal emerging health issues such as urinary tract infections in older adults [20].

#### Results for Parameters of Binary Occupancy

Data processing was performed on data collected from three users. Wake time and bedtime were estimated from the light intensity signal in each corresponding bedroom. Bedtime was defined as the last transition from light to dark occurring between 18:00 and 24:00 that was followed by at least 60 min of sustained darkness, whereas wake time was defined as the first transition from dark to light occurring between 03:00 and 12:00 that was followed by at least 60 min of sustained light.

Each continuous period of detected presence of at least 30 s in the bathroom is treated as a single visit. Visits occurring during bedtime are counted as nocturia episodes, while the remaining visits are counted as daytime bathroom visits.

**Figure 3:**
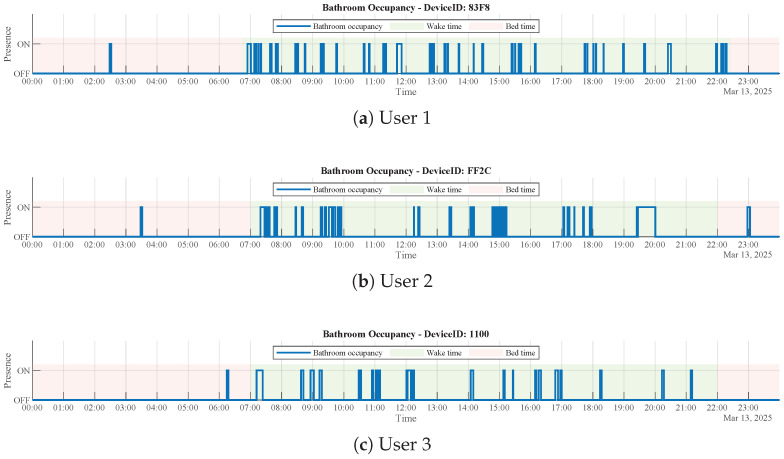
Bathroom occupancy results over a 24 h period for three different devices. (**a**) User 1 shows 1 nocturia event and 49 daytime visits. (**b**) User 2 shows 2 nocturia events and 52 daytime visits. (**c**) User 3 shows 1 nocturia event and 24 daytime visits.

Figure 3 shows the 24 h bathroom occupancy profiles for three different users, distinguishing between daytime visits and nocturia events. The results of these parameters are listed in Table 1.

### 3.2. Data Processing for mmWave Radar Energy Levels

Daily activity windows are segmented using a short-term average/long-term average (STA/LTA) detector applied to radar-derived energy levels. While conventional STA/LTA schemes often rely on static thresholds that require manual tuning and may degrade under non-stationary conditions, we adopt an adaptive thresholding approach inspired by ref. [21], where decision thresholds are derived from day-specific signal statistics. In our implementation, the STA/LTA window lengths are fixed, but the start/stop logic yields activity segments of variable duration and the thresholds adapt across users/days.

This section describes the adaptive STA/LTA detector used to segment bedroom activity into (i) bedroom occupancy from the micromovement energy signal and (ii) non-sedentary activity during occupancy from the macromovement energy signal. Moreover, we restrict the analysis to a predefined daytime window (wake-to-bed interval) to exclude sleep-related behavior, which is outside the scope of this work.

To simplify the analysis, and since occupancy detection relies on the occurrence of activity rather than its spatial location, all radar channels covering the 0–6 m range are aggregated into a single normalized activity signal. The aggregated radar energy signal C(k) is computed as follows:

(1) $$C\left(k\right)=\frac{1}{N}\sum_{j=1}^{N}\frac{c_{j}\left(k\right)}{100},$$

where $c_{j}\left(k\right)$ is the target energy at sample *k* in the *j*-th distance channel, and *N* is the number of channels. Aggregating channels in this way integrates reflected energy across range bins and provides a compact indicator of motion intensity within the monitored area. This is consistent with common practice in RF sensing, where energy-based metrics are used as a first step for motion segmentation in radar-based monitoring pipelines [21,22]. In this work, C(k) is computed from the micromovement energy for occupancy detection and from the macromovement energy for detecting non-sedentary activity during occupancy.

In practice, the aggregated radar energy signal C(k) is influenced by several factors beyond human motion, including thermal noise, static clutter, multipath, and slow environmental variations. These effects can introduce baseline drift and isolated fluctuations that may trigger false transitions if static thresholds are used. We mitigate them through a layered approach: (i) multi-range aggregation, which averages out channel-specific perturbations; (ii) normalization to a common bounded scale, improving day-to-day comparability; and (iii) STA/LTA moving-average windowing combined with a hysteresis scheme (separate activation/deactivation thresholds $\sigma_{1}$ and $\sigma_{3}$) to prevent rapid toggling around the baseline. Finally, the intermediate threshold $\sigma_{2}$ is set using a CFAR-like percentile rule computed from background-only samples, which adapts the decision boundary to the day-specific noise/clutter level. Together, these steps reduce sensitivity to impulsive spikes and slow drift while preserving sustained motion-related changes relevant for occupancy and non-sedentary activity detection.

#### 3.2.1. Formulation of the Adaptive STA/LTA Detector

The variable-window, ratio-based STA/LTA detector, as well as the start and stop conditions, are formulated as follows [21]:

(2) $$\mathrm{STA}\left(t\right)=\frac{1}{T_{1}}\sum_{k=t+1}^{t+T_{1}}C\left(k\right),\;\mathrm{LTA}\left(t\right)=\frac{1}{T_{2}}\sum_{k=t-T_{2}+1}^{t}C\left(k\right),$$

where $T_{1}$ and $T_{2}$ are the short- and long-term window lengths (in samples), and C(k) is the aggregated energy signal. The activation condition is defined as follows:

(3) $$\mathrm{STA}\left(t\right)>\sigma_{1}\;\mathrm{and}\;\frac{\mathrm{STA}(t)}{\mathrm{LTA}(t)}>\sigma_{2},$$

whereas deactivation occurs when

(4) $$\mathrm{STA}\left(t\right)<\sigma_{3}\;\mathrm{and}\;\frac{\mathrm{STA}(t)}{\mathrm{LTA}(t)}<\sigma_{2}.$$

Here, $\sigma_{1}$ is an absolute activation threshold that suppresses spurious detections during low-energy periods; $\sigma_{2}$ is the STA/LTA ratio threshold that governs activation when short-term energy rises substantially above the background level (and, conversely, deactivation when it falls back); and $\sigma_{3}$ is an absolute deactivation threshold that introduces hysteresis and prevents rapid toggling between active and inactive states. In general, $\sigma_{1}$ and $\sigma_{3}$ are tuned according to the signal statistics, whereas $\sigma_{2}$ primarily controls the sensitivity of the detector. The procedure used to compute the parameters of the adaptive STA/LTA detector is detailed in the following sections.

First, the STA/LTA parametrization for bedroom occupancy using the micromovement energy signal is described. Then, the same approach is used to detect non-sedentary activity from the macromovement energy signal.

In this work, the detector is adaptive in two complementary senses. First, although the STA and LTA window lengths ($T_{1}$ and $T_{2}$) are fixed, the start/stop logic in (3) and (4) yields activity segments whose duration adapts to the observed signal dynamics, allowing the method to capture events of variable length without manual retuning. Second, the decision thresholds $\left(\sigma_{1},\sigma_{2},\sigma_{3}\right)$ are computed automatically from the statistics of the STA/LTA signals on a per-day, per-user basis (and separately for micromovement and macromovement signals), so that the detector accounts for inter-user variability and day-to-day changes.

#### 3.2.2. Length of the STA and LTA

For the purpose of detecting bedroom presence from radar-based micromovement data sampled at 1 Hz, we considered a short-term window of $T_{1}\;=\;30$ s. This duration reflects the temporal scale of typical presence events, since occupancy in a room usually lasts at least several tens of seconds. This window length enables the detection of changes in motion intensity while maintaining temporal precision and avoiding excessive smoothing.

For the baseline, we set the long-term window to $T_{2}\;=\;300$ s, allowing the LTA to capture slow variations. Together with the short-term window of $T_{1}\;=\;30$ s, this configuration sets a ratio of $T_{2}/T_{1}\;=\;10$, which falls within the recommended range for STA/LTA detectors reported in ref. [23]. This setting enables the detector to distinguish transient micromovement activity from slow baseline variations, providing stable performance for continuous detection of events [21,22].

#### 3.2.3. Activation Threshold, $\sigma_{1}$, and Deactivation Threshold, $\sigma_{3}$

**Figure 4:**
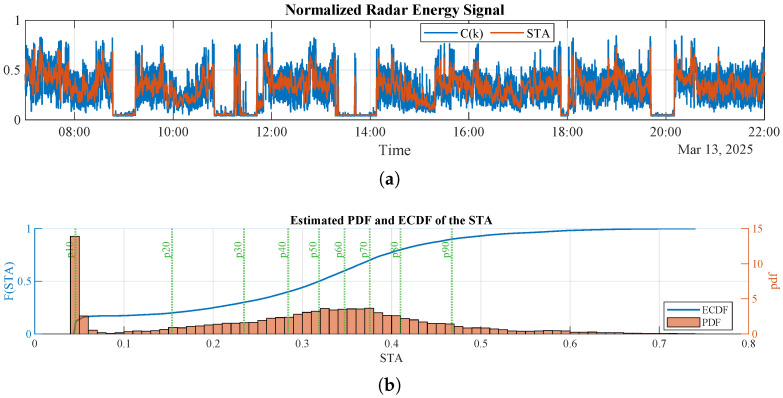
Micromovement radar energy signal during daytime and its STA: (**a**) normalized micromovement radar energy signal C(k) and the corresponding STA computed with $T_{1}=30$ s. (**b**) estimated CDF and PDF of the STA, where low values represent the non-occupancy baseline; $\sigma_{1}$ and $\sigma_{3}$ are derived from these distributions.

To determine suitable threshold selection criteria, it is important to characterize the statistical properties of the STA of the radar energy signal C(k). In this regard, Figure 4 shows the radar energy signal C(k) recorded during daytime hours in one of the bedrooms and its corresponding STA computed with $T_{1}=30$ s. The figure also includes the empirical cumulative distribution function (ECDF) of the STA and a nonparametric estimate of its probability density function (PDF). As can be seen, over a one-day window the STA, and therefore the radar energy signal, follow a non-normal distribution, with a significant accumulation of samples at low STA values; this property is exploited to derive the deactivation threshold $\sigma_{3}$.

The PDF of the STA depends on both the level of user activity inside the room, as captured by the radar energy signal, and the daytime occupancy percentage, and it varies from day to day and across users. Therefore, adaptive thresholds are computed from the statistics of the signal.

From this perspective, the activation threshold for the STA, $\sigma_{1}$, is defined using a nonparametric statistics approach (i.e., percentiles), as reported in ref. [24]:

(5) $$\sigma_{1}=p_{75}\left(\mathrm{STA}\right)$$

To set the STA deactivation threshold $\sigma_{3}$, we employ a relative-slope criterion. It is assumed that non-occupancy time windows in the STA are characterized by low STA values. Accordingly, $\sigma_{3}$ is defined as the first STA value at which the derivative of the ECDF falls below a fixed fraction, α, of its maximum, defined in (6) and (7).

(6) $$M\;=\;\max_{\mathrm{STA}}\frac{dF(\mathrm{STA})}{d\;\mathrm{STA}}$$

(7) $$\sigma_{3}\;=\;\min_{\mathrm{STA}}\left\{\frac{dF(\mathrm{STA})}{d\;\mathrm{STA}}\;\leq \;\alpha \cdot M\right\}$$

where *F* is the ECDF, and α=0.2 corresponds to the point where the ECDF slope decreases to 20% of its peak.

The implemented approach belongs to the family of first-derivative cutoff methods [25], where the slope fraction α is a tunable parameter controlling sensitivity. Its use is consistent with derivative-threshold (DFDT) approaches commonly described in the literature [26].

**Figure 5:**
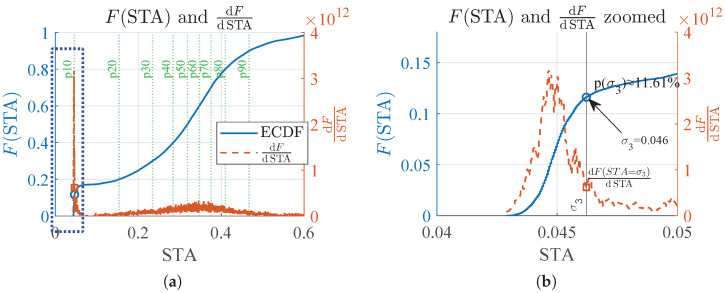
Application of the relative-slope criterion for the computation of $\sigma_{3}$. Panel (**b**) shows the resulting value of $\sigma_{3}$ and its corresponding STA percentile. (**a**) ECDF and its derivative as a function of STA. (**b**) Zoomed view of the blue rectangle in (**a**).

Figure 5 illustrates the procedure for using the relative-slope criterion to compute the deactivation threshold $\sigma_{3}$. It can be seen that, with a value of α=0.2, the resulting $\sigma_{3}$ ensures that deactivation occurs at STA levels that remain slightly above the non-occupancy baseline, since data corresponding to this baseline are concentrated at low STA values, consistent with the ECDF shown in Figure 4.

#### 3.2.4. Sensitivity Threshold, $\sigma_{2}$

The sensitivity threshold, $\sigma_{2}$, is set using a percentile constant false alarm rate (CFAR) rule on r=STA/LTA. First, a background set B is built by placing temporal guard bands around regions with elevated STA ($\mathrm{STA}>\sigma_{1}$) and by retaining only low-energy frames ($\mathrm{STA}<\sigma_{3}$). The sensitivity threshold $\sigma_{2}$ is set to the *q*-th percentile of the background ratio r=STA/LTA with $q=100(1-P_{\mathrm{FA}})\%$, so that only a fraction $P_{\mathrm{FA}}$ of background samples exceed the threshold (false alarms). Following standard CFAR practice, the percentile is tied to a specific false-alarm rate $P_{\mathrm{FA}}$; in this work, it is set as $P_{\mathrm{FA}}=10^{-3}$, a practical operating point reported in [27,28,29]. Conceptually, this matches a percentile/order statistics constant false alarm rate (OS-CFAR) thresholding of the STA/LTA ratio using a sliding reference window with guard cells, a standard practice in CFAR design [27,30].

#### 3.2.5. Adaptive Parameter Update Procedure and Implementation Considerations

Algorithm 1 summarizes the adaptive procedure used to compute the STA/LTA thresholds from daily radar-energy statistics and to segment the signal into activity windows. The adaptation is not an iterative optimization. Instead, for each radar unit and each analyzed day (or segment), the thresholds are recomputed once from the corresponding STA/LTA statistics, making the procedure capable of capturing day-to-day and inter-user variability.
**Algorithm 1** Adaptive STA/LTA detector: parameter adaptation and segmentation**Require:** Aggregated radar energy C(k); sampling rate $f_{s}$; window lengths $T_{1}$ (STA), $T_{2}$
      (LTA); STA percentile $p_{75}$ (for $\sigma_{1}$); slope fraction α (for $\sigma_{3}$); false-alarm target $P_{\mathrm{FA}}$ and
      guard time $T_{g}$ (for $\sigma_{2}$)
**Ensure:** Activity mask m(k)
  1: Compute STA and LTA over $T_{1}$ and $T_{2}$, and the ratio r(k)=STA(k)/LTA(k)
  2: Set $\sigma_{1}$ as the $p_{75}$-th percentile of STA (day/user-specific).
  3: Set $\sigma_{3}$ from the ECDF of STA using a relative-slope rule (first STA value where slope
     ≤α times the peak slope).
  4: Estimate $\sigma_{2}$ from background-only samples: exclude $\pm T_{g}$ around candidate activity
     periods and set a CFAR-like percentile using $P_{\mathrm{FA}}$.
  5: Segment with hysteresis: start when (STA $>\sigma_{1}$)and (ratio $>\sigma_{2}$); stop when (STA $<\sigma_{3}$)
     and (ratio $<\sigma_{2}$).
  6: Output m(k).


Additionally, to make the adaptive STA/LTA procedure well-defined across users and days, the following rules are applied.

- Minimum data length: If the available record (day/segment) is too short to reliably estimate the long-term baseline (e.g., the duration is less than $T_{2}$ or if there is an insufficient number of samples), thresholds are not updated and the segment/day is excluded from further analysis.
- Numerical stability of the ratio: The STA/LTA ratio is computed as follows:
  $$r\left(k\right)=\frac{\mathrm{STA}(k)}{\max(\mathrm{LTA}(k),\varepsilon )},\;\varepsilon =10^{-6},$$
  to avoid numerical issues when LTA(k) is close to zero.
- ECDF-rule fallback for $\sigma_{3}$: If the ECDF relative-slope rule does not yield a valid $\sigma_{3}$ (e.g., no drop below α of the peak slope is found), $\sigma_{3}$ is set using a conservative low-percentile fallback (e.g., $\sigma_{3}=p_{15}\left(\mathrm{STA}\right)$), which represents the non-occupancy baseline.
- Insufficient background for CFAR-like $\sigma_{2}$: $\sigma_{2}$ is estimated as a CFAR-like percentile of the ratio r(k) computed on *background-only* samples, after excluding a guard interval of $\pm T_{g}$ around candidate activity periods. In practice, background samples are selected using the low-energy condition $\mathrm{STA}\left(k\right)<\sigma_{3}$. If this yields too few samples to obtain a stable percentile estimate, we relax the background selection (while keeping the guard exclusion) by using a more permissive limit:
  $$\mathrm{STA}\left(k\right)<\max\left(\sigma_{3},\;p_{35}\left(\mathrm{STA}\right)\right).$$
  If the background set remains insufficient, $\sigma_{2}$ is not updated for that segment/day.
- Bounding $\sigma_{2}$ and edge handling: To prevent extreme sensitivity on atypical days, $\sigma_{2}$ is clipped to a bounded range, $\sigma_{2}\in \left[\sigma_{2,\min},\;\sigma_{2,\max}\right]$ (in our implementation, $\sigma_{2,\max}=10$ and $\sigma_{2,\min}=0.5$). Moreover, STA and LTA are computed using finite-length moving averages; near the beginning/end of the record the effective window is shortened so that no samples outside the available data are used.
- Hysteresis consistency: If the adaptive thresholds yield $\sigma_{3}\geq \sigma_{1}$ (degenerate hysteresis), we enforce a minimum separation by resetting
  $$\sigma_{3}\leftarrow \max\left(\sigma_{\min},\sigma_{1}-\Delta \right),\;\sigma_{\min}=0,\;\Delta =0.05.$$
  This guarantees $\sigma_{3}<\sigma_{1}$ and prevents unstable toggling.
- Per-user/per-scenario adaptation: The STA/LTA thresholds are updated independently for each radar unit (i.e., per room and per user) and for each analyzed day/segment. In practice, $\sigma_{1}$ and $\sigma_{3}$ are derived from the day-specific STA distribution, while $\sigma_{2}$ is estimated from day-specific background-only samples using the CFAR-like rule. This makes the detector automatically adapt to different occupants (e.g., lower-amplitude vs. more active users) and to scenario-dependent propagation conditions (e.g., sensor placement, room geometry, clutter), without manual retuning.

#### 3.2.6. Results of Bedroom Occupancy Detection

**Figure 6:**
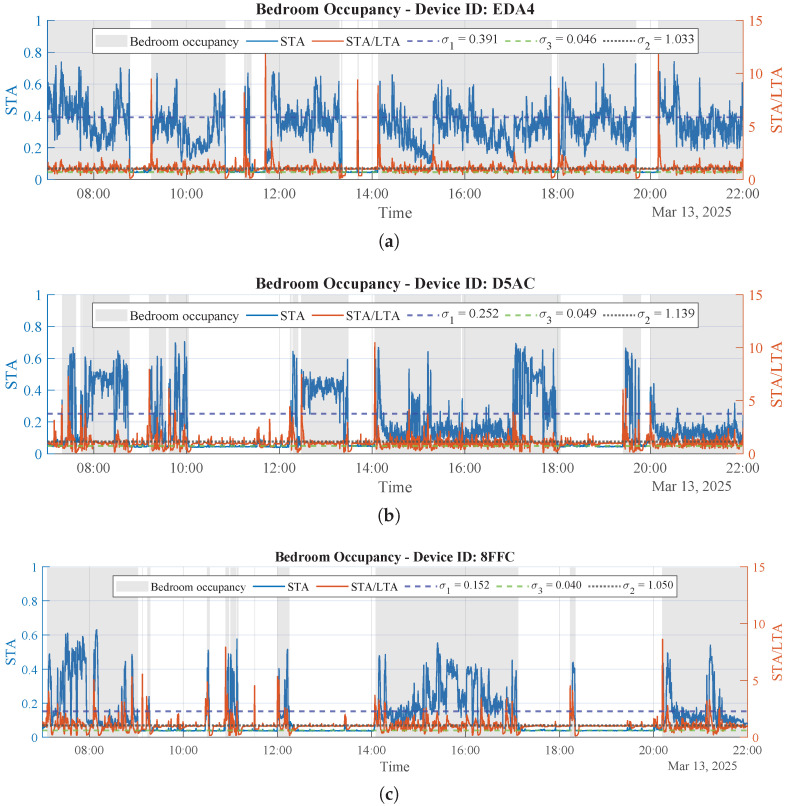
Bedroom occupancy estimated from the micromovement radar signal using the proposed adaptive STA/LTA detector for the three monitored users. Gray zones indicate detected occupancy. (**a**) Bedroom occupancy for User 1 obtained with the proposed adaptive STA/LTA detector. (**b**) Bedroom occupancy for User 2 obtained with the proposed adaptive STA/LTA detector. (**c**) Bedroom occupancy for User 3 obtained with the proposed adaptive STA/LTA detector.

Figure 6 shows the results of using the proposed adaptive STA/LTA approach to obtain bedroom occupancy from the micromovement radar signal, with shaded intervals indicating periods classified as occupied according to the start and stop conditions defined in Equations (3) and (4). Results are shown for three different users (different device IDs) on the same day. As can be seen, for fixed values of $T_{1}=30$ and $T_{2}=300$, the adaptive approach computes $\sigma_{1}$, $\sigma_{2}$, and $\sigma_{3}$ from the corresponding signal statistics for each user, as summarized in Table 2.

To support the use of the adaptive STA/LTA detector beyond the illustrative cases in this section, Appendix A reports additional qualitative examples (5 different days for two users), illustrating stable occupancy detection across days with different STA statistics.

#### 3.2.7. Comparison vs. a Fixed-Threshold Baseline

To assess the benefit of the proposed adaptive thresholding, we performed a diagnostic comparison against a fixed STA/LTA configuration using a simple manual reference. For each user/day shown in Figure 6, we manually annotated bedroom-presence intervals by inspecting the STA time series and marking contiguous segments with sustained activity above the background level. This manual annotation is used as an indicative reference for segmentation behavior.

We evaluate both the proposed adaptive method and the fixed-threshold baseline against the manual annotation over the 07:00–22:00 window using two diagnostic metrics: (i) $\Delta T_{\mathrm{occ}}=T_{\mathrm{occ}}-T_{\mathrm{occ}}^{\mathrm{manual}}$ (minutes), i.e., the deviation in total detected occupied time with respect to the manual annotation; and (ii) $N_{\mathrm{trans}}$, the number of ON ↔ OFF transitions, which is informative of overly “sticky” segmentations (too few transitions) or unstable toggling (too many transitions). Here, $T_{\mathrm{occ}}$ denotes the total time labeled as occupied (ON) by the considered method within 07:00–22:00, and $T_{\mathrm{occ}}^{\mathrm{manual}}$ denotes the corresponding total occupied time from the manual STA-based annotation.

For the fixed-threshold baseline, we keep the same STA/LTA windows ($T_{1}$ and $T_{2}$), the same preprocessing, and the same hysteresis start/stop logic as in the proposed detector, but we use constant thresholds that are identical for all users and days:

$$\sigma_{1}^{\mathrm{fix}}=0.25,\;\sigma_{3}^{\mathrm{fix}}=0.08,\;\sigma_{2}^{\mathrm{fix}}=1.5.$$

These values were selected once as conservative operating points assuming the STA is normalized to [0,1] and preserving the intended hysteresis ordering ($\sigma_{3}^{\mathrm{fix}}<\sigma_{1}^{\mathrm{fix}}$).

Table 3 shows that the fixed-threshold baseline yields similar results to the adaptive method for Users 2–3 on this specific day, suggesting that a single global setting can be acceptable when the STA statistics remain relatively stable. In contrast, for User 1 the fixed setting produces a much larger occupancy mismatch ($\Delta T_{\mathrm{occ}}=-120$ min) and many more ON↔OFF transitions ($N_{\mathrm{trans}}=50$), indicating inadequate activation/deactivation under a different STA distribution. Overall, these results support adapting thresholds per user/day to cope with inter-user variability and day-to-day changes in radar-energy statistics.

#### 3.2.8. Results of Non-Sedentary Behavior During Bedroom Occupancy

In the context of room monitoring and physical activity classification, we assume that a person can either be in a stationary (sedentary) state or performing non-sedentary activities. In clinical and epidemiological terms, room activity is classified as sedentary (≤1.5 metabolic equivalents of task, METs, while sitting, reclining, or lying) [31], light-intensity physical activity (LPA; 1.6–2.9 METs, typically involving slow ambulation, slow walking, and routine household tasks), or moderate-intensity activity (MIA; ≥3 METs, such as making the bed with a linen change, organizing a room, or bending and lifting light items) [32]. From this perspective, given that macromovement sensing targets whole-body displacements (e.g., walking or bed making), the adaptive STA/LTA detector described in Section 3.2 is applied to determine periods of non-sedentary activity (i.e., LPA or MIA).

These results, combined with bedroom occupancy, provide an estimate of how active the person is while staying in the bedroom. This information is particularly valuable for non-intrusive indoor monitoring of older adults [33]. In this regard, international guidelines emphasize reducing sedentary time and increasing physical activity of any intensity in older adults, which motivates distinguishing sedentary periods from LPA and moderate activity during bedroom occupancy [31]. Moreover, spending more time in LPA relative to sedentary time is associated with a more favorable frailty profile [34] and with greater odds of healthy aging [35].

**Figure 7:**
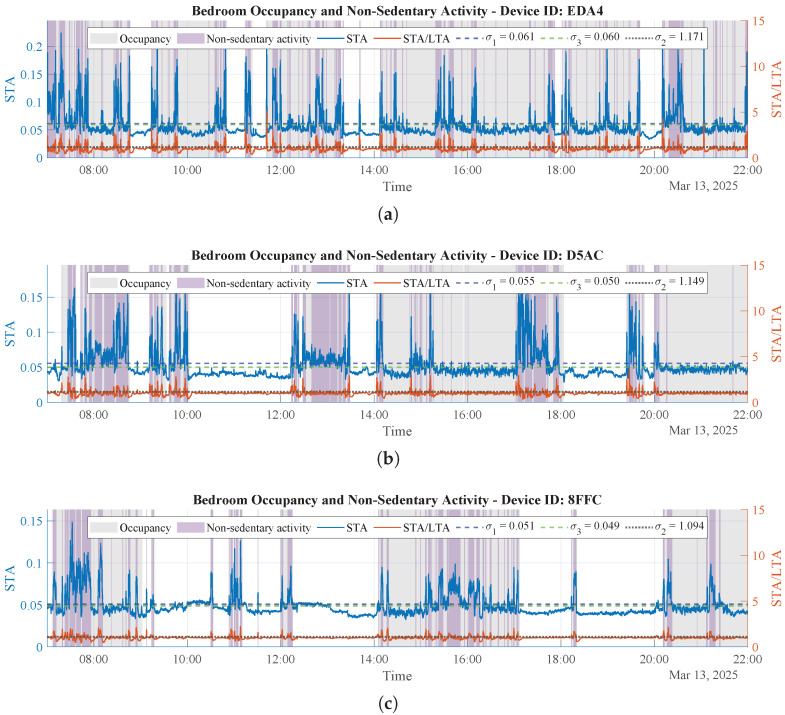
Physical activity detected during bedroom occupancy for three users using the STA/LTA algorithm applied to macromovement data. Gray zones indicate bedroom occupancy, whereas purple zones indicate non-sedentary activity periods. (**a**) Percent of bedroom-occupied time in non-sedentary activity of User 1. (**b**) Percent of bedroom-occupied time in non-sedentary activity of User 2. (**c**) Percent of bedroom-occupied time in non-sedentary activity of User 3.

Figure 7 shows the results of detecting non-sedentary activity during bedroom occupancy for three different users (different device IDs). These results are combined with bedroom occupancy detection to estimate the percentage of bedroom-occupied time spent in non-sedentary activity. These data enable estimation of how sedentary a person is while staying in the bedroom, a clinically relevant indicator given the links between sedentary behavior, light-intensity activity, and health outcomes in older adults [31,34,35]. Table 4 summarizes, for each monitored user, the computed percentage of daytime spent in the bedroom and the percentage of bedroom-occupied time classified as non-sedentary activity.

It is worth noting that the activation and deactivation thresholds shown in Figure 6 and Figure 7 differ. Although the signals correspond to the same user, the statistics of the micromovement data (used for occupancy detection) differ from those of the macromovement data (used for non-sedentary activity detection). As a consequence, the thresholds change, reflecting the adaptive nature of the proposed procedure for computing these parameters.

## 4. Processing of UWB Positioning Data

UWB positioning data consist of *x*, *y*, and *z* coordinates together with a timestamp and are used to evaluate mobility-related well-being parameters in common areas.

In general, the Qorvo DWM1001 UWB real-time location system (RTLS) reports that the typical X–Y location accuracy under line-of-sight (LOS) conditions is around 10 cm [17]; however, precision decreases in non-line-of-sight (NLOS) conditions [36]. From this perspective, to overcome possible outliers that lead trajectories to pass through walls, and increased position errors in non-line-of-sight (NLOS) zones such as the kitchen, hall, and corridors, a partially constrained Kalman filter (KF) approach has been implemented. This approach, reported in [37], enables trajectory smoothing and correction using map description as additional information.

### 4.1. UWB Data Preprocessing

Before applying the partially constrained Kalman filter, raw UWB positions are preprocessed using the available map information (zones, doors, corridors, hall, and forbidden regions). The goal is to obtain a trajectory that is both temporally consistent and physically feasible with respect to the indoor layout, so that the subsequent filtering step is not forced to explain impossible jumps (e.g., crossing walls) or artefactual samples in forbidden areas.

**Figure 8:**
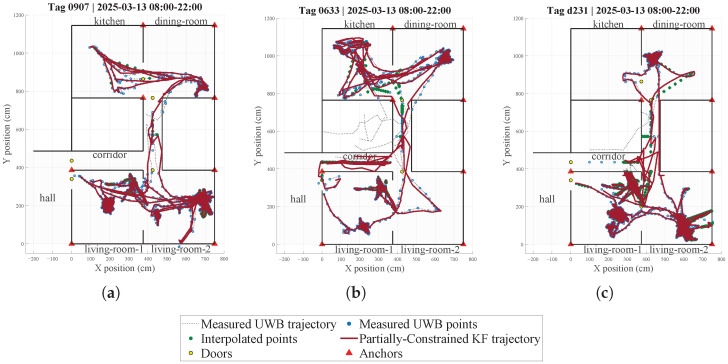
Daily trajectories of three users in common areas: (**a**) User 1, (**b**) User 2, and (**c**) User 3, smoothed using the partially constrained Kalman filter approach.

First, samples lying in forbidden rooms near corridors are moved vertically or horizontally to the closest feasible corridor. Then, short temporal gaps (duration <60 s), which occur primarily during corridor transitions, are filled. If the straight line between the gap endpoints does not intersect any wall or forbidden zone, intermediate samples are generated by linear interpolation; otherwise, a polyline path is constructed through the sequence of doors connecting the corresponding zones, so that transitions such as *hall*→ *dining-room* occur through a valid doorway instead of crossing walls. Door transitions are enforced using predefined door anchor points located at the midpoint of each doorway (Figure 8), which restricts gap filling to feasible zone-to-zone transitions.

All interpolated samples are subsequently assigned an inflated measurement covariance. Finally, samples lying inside corridor polygons are snapped to the geometric center line defined by the surrounding walls. The resulting preprocessed trajectory is thus physically consistent with the indoor map and is used as input to the partially constrained NCV Kalman filter.

UWB localization errors are typically low under line-of-sight conditions but increase under NLOS conditions, producing intermittent outliers. Since no centimeter-level ground truth is available in the residence, we do not report absolute positioning accuracy. Instead, we limit the impact of NLOS/outliers on mobility indicators by combining: (i) map-based preprocessing (projection of samples in forbidden regions onto feasible corridors and topology-consistent short-gap filling), (ii) inflated measurement covariance for interpolated samples, and (iii) an event-driven wall-crossing check that triggers a partially constrained KF correction only when a physically impossible transition is detected. Longer gaps are treated as missing coverage and excluded from time-based mobility summaries; therefore, mobility indices are computed only over valid UWB-coverage intervals using the actual inter-sample durations. In our computations, missing data reduce the total monitored time, which is reported for each user/day.

### 4.2. Description of 2D Linear Partially Constrained KF

The sequence of positions is filtered using a linear Kalman filter with a 2D nearly constant velocity (NCV) model to represent approximately uniform motion of people in indoor tracking. For this purpose, the state vector at time step *k* is defined as follows:

(8) $$X_{k}=\left[\begin{array}{c} x_{k}\\ y_{k}\\ v_{x,k}\\ v_{y,k} \end{array}\right],$$

where $\left(x_{k},y_{k}\right)$ is the position (in cm) and $\left(v_{x,k},v_{y,k}\right)$ are the corresponding velocities (in cm/s).

For a fixed sampling period $T_{s}$, the NCV dynamics are modeled as follows:

(9) $$x_{k}=A\cdot x_{k-1}+w_{k},$$

with a time-invariant state transition matrix

(10) $$A=\left[\begin{array}{cccc} 1 & 0 & T_{s} & 0\\ 0 & 1 & 0 & T_{s}\\ 0 & 0 & 1 & 0\\ 0 & 0 & 0 & 1 \end{array}\right],$$

where $w_{k}∼N\left(0,Q\right)$ is a zero-mean Gaussian process noise. A 2D nearly constant velocity (NCV) model with white-acceleration process noise was used for the state dynamics, following standard formulations in target tracking [38].

(11) $$Q=q_{\mathrm{proc}}\cdot \left[\begin{array}{cccc} \frac{T_{s}^{3}}{3} & 0 & \frac{T_{s}^{2}}{2} & 0\\ 0 & \frac{T_{s}^{3}}{3} & 0 & \frac{T_{s}^{2}}{2}\\ \frac{T_{s}^{2}}{2} & 0 & T_{s} & 0\\ 0 & \frac{T_{s}^{2}}{2} & 0 & T_{s} \end{array}\right].$$

The NCV process noise intensity $q_{\mathrm{proc}}$ controls how much the velocity is allowed to change between consecutive time steps. For a sampling period $T_{s}$, the variance in the velocity increment per time step in each axis is $\sigma_{\Delta v}^{2}=q_{\mathrm{proc}}\cdot T_{s}$, so that the corresponding standard deviation is $\sigma_{\Delta v}=\sqrt{q_{\mathrm{proc}}\cdot T_{s}}$. In this work, $q_{\mathrm{proc}}$ was set to $100\;{\mathrm{cm}}^{2}{/s}^{3}$, which for $T_{s}=1\;s$ yields $\sigma_{\Delta v}\approx 10\;\mathrm{cm}/s$. This corresponds to smooth indoor motion for an older adult walking at about 40–60cm/s, allowing for moderate step-to-step changes in speed while still smoothing the estimated trajectory.

The measurement model only observes the (x,y) components of the state, which correspond to UWB positioning data; it is defined as follows:

(12) $$z_{k}=H\cdot X_{k}+v_{k},$$

with

(13) $$H=\left[\begin{array}{cccc} 1 & 0 & 0 & 0\\ 0 & 1 & 0 & 0 \end{array}\right],$$

and Gaussian noise $v_{k}∼N\left(0,R_{k}\right)$. For direct UWB measurements, a nominal measurement covariance matrix *R* is used. In line-of-sight (LOS) conditions, we model the position noise as independent in *x* and *y* with $\sigma_{x}=\sigma_{y}=10\;\mathrm{cm}$. This value is consistent with the typical LOS positioning accuracy specified for the DWM1001 module and is used as the nominal measurement standard deviation in the Kalman filter [17]:

(14) $$R=\left[\begin{array}{cc} \sigma_{x}^{2} & 0\\ 0 & \sigma_{y}^{2} \end{array}\right].$$

Whereas for interpolated points, an inflated covariance is used to reflect their lower reliability, $\alpha_{R}\cdot R$. In our implementation, we set $\alpha_{R}=4$, which multiplies the measurement variance by a factor of four and thus doubles the corresponding standard deviation.

#### 4.2.1. Initialization

For each continuous time segment of data, the filter is initialized with the first valid position as follows:

(15) $$X_{k_{0}}=\left[\begin{array}{c} x_{k_{0}}\\ y_{k_{0}}\\ 0\\ 0 \end{array}\right],\;P_{k_{0}}=\mathrm{diag}\left(\sigma_{x,0}^{2},\sigma_{y,0}^{2},\sigma_{v_{x},0}^{2},\sigma_{v_{y},0}^{2}\right),$$

where $P_{k_{0}}$ is the initial state covariance matrix.

#### 4.2.2. Prediction

The predicted state and covariance are computed as follows:

(16) $$X_{k}^{-}=A\cdot {\hat{X}}_{k-1},$$

(17) $$P_{k}^{-}=A\cdot P_{k-1}\cdot A^{⊤}+Q.$$

#### 4.2.3. Measurement Update (Unconstrained KF)

Given the predicted state estimate $X_{k}^{-}$ and covariance $P_{k}^{-}$, the KF measurement update is applied using UWB position measurement data $z_{k}$. The innovation is:

(18) $$V_{k}=z_{k}-H\cdot X_{k}^{-},$$

with innovation covariance

(19) $$S_{k}=H\cdot P_{k}^{-}\cdot H^{⊤}+R_{k},$$

and (unconstrained) Kalman gain

(20) $$K_{k}=P_{k}^{-}\cdot H^{⊤}\cdot S_{k}^{-1}.$$

The unconstrained updated state and covariance, denoted as ${\hat{X}}_{k}$ and $P_{k}$, are computed as follows:

(21) $${\hat{X}}_{k}=X_{k}^{-}+K_{k}\cdot V_{k},$$

(22) $$P_{k}=\left(I-K_{k}\cdot H\right)\cdot P_{k}^{-}.$$

#### 4.2.4. Detection of Wall Crossings and Constrained State

The map information is represented as a set of horizontal and vertical walls, modeled as line segments in the (x,y) plane. At each time step *k*, after computing the unconstrained updated state ${\hat{X}}_{k}$ from the KF, we consider the line segment joining the posterior state at time k−1, ${\hat{X}}_{k-1}$, and the unconstrained updated state at time *k*, ${\hat{X}}_{k}$, and test whether it intersects any wall segment. If no wall crossing is detected, the unconstrained update is kept. Otherwise, the map information is used to construct a geometrically feasible target position $\left(x_{\mathrm{tgt}},y_{\mathrm{tgt}}\right)$ on or slightly inside the corresponding wall, using a small safety offset to remain on the feasible side.

This target is set as a linear equality constraint on the state vector. For a horizontal wall, only the *y*-coordinate is constrained:

(23) $$C=\left[\begin{array}{cccc} 0 & 1 & 0 & 0 \end{array}\right],\;d=y_{\mathrm{tgt}}.$$

For a vertical wall, only the *x*-coordinate is constrained:

(24) $$C=\left[\begin{array}{cccc} 1 & 0 & 0 & 0 \end{array}\right],\;d=x_{\mathrm{tgt}},$$

and when both a horizontal and a vertical wall are crossed simultaneously:

(25) $$C=\left[\begin{array}{cccc} 1 & 0 & 0 & 0\\ 0 & 1 & 0 & 0 \end{array}\right],\;d=\left[\begin{array}{c} x_{\mathrm{tgt}}\\ y_{\mathrm{tgt}} \end{array}\right].$$

Each detected wall crossing thus leads to a linear equality constraint of the form:

(26) $$C\cdot {\tilde{X}}_{k}=d,$$

where ${\tilde{X}}_{k}$ is the constrained updated state at time *k*, obtained by combining the unconstrained KF update with the partially constrained formulation reported in ref. [37].

#### 4.2.5. Partially Constrained Kalman Update

Starting from the unconstrained KF gain, $K_{k}$, consistency between the measurement update and the constrained state ${\tilde{X}}_{k}$ is enforced by computing a modified gain ${\tilde{K}}_{k}$ using an analytical method based on Lagrange multipliers:

(27) $$\begin{array}{cc} {\tilde{K}}_{k} & =K_{k}-D^{⊤}\cdot {\left(D\cdot D^{⊤}\right)}^{-1}\cdot \left(D\cdot {\hat{X}}_{k}-{\tilde{X}}_{k}\right)\\ \; & \;\cdot {\left(V_{k}^{⊤}\cdot S_{k}^{-1}\cdot V_{k}\right)}^{-1}\cdot V_{k}^{⊤}\cdot S_{k}^{-1}, \end{array}$$

where $D=I_{4}$. The final, partially constrained state estimate is then obtained as follows:

(28) $${\hat{X}}_{k}=X_{k}^{-}+{\tilde{K}}_{k}\cdot V_{k}.$$

The associated covariance update is also computed:

(29) $$\begin{array}{cc} P_{k} & =P_{k}^{-}-P_{k}^{-}\cdot H^{⊤}\cdot {\tilde{K}}_{k}^{⊤}-{\tilde{K}}_{k}\cdot H\cdot P_{k}^{-}\\ \; & \;+{\tilde{K}}_{k}\cdot \left(H\cdot P_{k}^{-}\cdot H^{⊤}\right)\cdot {\tilde{K}}_{k}^{⊤}+{\tilde{K}}_{k}\cdot R_{k}\cdot {\tilde{K}}_{k}^{⊤}. \end{array}$$

**Implementation details and computational aspects.** In this work, all UWB trajectory processing is performed offline. The map is encoded as a set of axis-aligned wall segments (horizontal/vertical line segments in the (x,y) plane) and valid/forbidden area polygons. Map information is quantified as linear equality constraints on the NCV state, applied only to the position components: for a vertical wall we enforce $x=x_{\mathrm{tgt}}$ with C=[1000], $d=x_{\mathrm{tgt}}$; for a horizontal wall we enforce $y=y_{\mathrm{tgt}}$ with C=[0100], $d=y_{\mathrm{tgt}}$ (and both rows are stacked if needed). Constraints are integrated event-wise: at each step we run the unconstrained KF update, test whether the segment joining consecutive posterior positions intersects any wall segment, and only in that case compute $\left(x_{\mathrm{tgt}},y_{\mathrm{tgt}}\right)$ on the feasible side (with a small safety offset) and apply the partially constrained update. The added cost is dominated by segment–segment intersection checks against *W* wall segments (i.e., O(W) per sample), whereas the constrained correction involves only small (4D) matrix operations. Consistency of short-gap filling with indoor topology and plausible movements is ensured by the preprocessing rules described in Section 4.1; in particular, door-to-door transitions are enforced by routing the interpolated polyline through predefined door anchor points located at the midpoint of each doorway, as illustrated in Figure 8.

### 4.3. Results of Processing UWB Data

Figure 8 shows the daily trajectories for three different users, obtained from UWB data processed with the partially constrained KF approach. The red line represents the UWB data smoothed by the partially constrained KF, while blue dots denote the raw UWB measurements and the dotted black line their unconstrained interpolated trajectory. As can be seen, the filter smooths the trajectory and projects measurements falling in forbidden zones onto the nearest corridor, thus enforcing the constraints while preserving the overall path shape.

From the partially constrained KF trajectories (red line in Figure 8), we derive a set of daily mobility parameters in the context of aging and frailty. Instantaneous walking speed is computed as v(t)=∥v(t)∥; from this, two distribution-based metrics are obtained: (i) habitual gait speed (median of all walking time windows) and (ii) maximum gait speed (95th percentile). Both parameters are established predictors of survival, disability, and frailty in older adults [39,40], and recent work highlights the value of free-living, distribution-based gait metrics obtained from wearable sensors [41].

Moreover, daily walked distance is estimated by integrating consecutive displacements of the filtered trajectory, restricted to samples above a low-speed threshold. Walking volume has been associated with reduced morbidity and mortality in epidemiological cohorts [42]. We also compute active vs. sedentary time by thresholding v(t), since the balance between sedentary behavior and light-to-moderate activity is linked to cognitive and functional outcomes in older adults [43].

Note that from these data, room-level semantic mapping of positions can be derived (e.g., living rooms, dining room, kitchen, corridor). Although we do not derive activities of daily living (ADL) indicators or routine-pattern metrics in the present work, these UWB-based occupancy data could be used in future studies to quantify time spent per room, transitions between zones, and routine stability, in line with the type of analyses reported in ref. [44].

Table 5 summarizes the UWB-derived mobility metrics computed from positions and velocities estimated along the trajectories obtained with the partially constrained KF described in Section 4.2. Active and sedentary time were computed using the actual inter-sample intervals and a speed threshold of 0.2 m/s (active for v>0.2 m/s), while intervals separated by temporal gaps longer than 5 min were treated as missing and therefore did not contribute to the time totals. Consequently, the total monitored time corresponds to the amount of valid UWB data available within the 8–22 h window rather than the full observation period. Habitual and maximum gait speed mainly reflect short transitions between adjacent zones in the monitored common areas, since active time is relatively low compared with sedentary time and corresponds primarily to brief walking bouts between locations, whereas the person otherwise remains in the living room or is seated during meals. In addition, time spent in the charging area was excluded from sedentary time when the tag remained within the charging zone for at least 5 min with low speed (v<0.2 m/s), to avoid overestimating inactivity due to device charging. Future work will investigate aggregating these parameters into weekly and monthly summaries to provide actionable insights for healthcare personnel.

## 5. Conclusions

The present work presents a practical case of monitoring older adults using mmWave radar and UWB technologies. mmWave radar is used in a non-intrusive, device-free manner, with one device installed in each user’s bathroom and bedroom. Relative-strength signals obtained from the mmWave radar module enable the classification of micromovements and macromovements into bedroom occupancy and non-sedentary activity.

For this purpose, an adaptive STA/LTA detector has been implemented, using an aggregated, normalized energy signal as input. Beyond applying the detector, a methodology for selecting and computing the STA/LTA window lengths and thresholds tailored to this type of mmWave radar signal has been proposed, combining nonparametric statistics, ECDF-based thresholding, and CFAR-inspired criteria. This adaptive design avoids manual tuning of detector parameters by relying on per-day, per-user statistics, which is important given the variability in bedroom occupancy patterns and activity levels among older adults.

In parallel, a UWB tag-based positioning system has been implemented to track mobility in common areas and to estimate daily gait and mobility metrics. For this purpose, a partially constrained Kalman filter approach using a nearly constant velocity (NCV) dynamics model has been used. This implementation leads to trajectory smoothing and trajectory correction using map information, especially in areas with non-line-of-sight (NLOS) conditions between tags and anchors, resulting in smoothed trajectories that respect the floor-plan constraints.

Together, these radar- and UWB-derived indicators illustrate how minimally intrusive RF monitoring (device-free in private rooms and tag-based in shared areas) can provide personalized information on bedroom occupancy, sedentary behavior, and mobility in shared spaces for older adults.

Although several of the extracted indicators are clinically motivated (e.g., gait-speed distribution metrics and active/sedentary time are widely used proxies of physical function), the present work is not a clinical validation study and our dataset does not include concurrent clinical assessments (frailty scores, cognitive tests, or clinical outcomes) to quantify such associations. Therefore, we restrict our claims to the feasibility and reproducibility of the proposed processing pipeline and the resulting digital mobility/occupancy markers.

The system was designed for real residential environments and therefore comes with typical deployment considerations. Coverage requirements imply installing mmWave nodes in private rooms and multiple UWB anchors in shared areas. Since UWB is tag-based, wearing/charging adherence affects the amount of usable data; accordingly, our pipeline treats long gaps as missing coverage rather than inactivity; so, incomplete adherence primarily reduces total monitored time. Moreover, while RF monitoring avoids cameras and does not capture visual content, the derived location- and routine-related traces should still be handled under standard data-governance practices (access control, minimization, retention). Finally, operating in NLOS/multipath conditions may introduce residual uncertainty; addressing this rigorously would require synchronized reference measurements and (when clinically relevant) concurrent assessments.

Potential optimizations include: (i) focusing deployment on the rooms/areas required by the care objective to reduce installation and maintenance effort; (ii) exploring UWB layouts with fewer anchors and/or lightweight calibration strategies to lower infrastructure requirements; and (iii) improving tag adherence via longer-battery wearables and simple staff-supported charging routines. From a privacy perspective, data exposure can be reduced by storing only aggregated daily indicators (instead of raw trajectories) when sufficient for the application, together with strict data-retention limits and role-based access control.

Potential future work includes validating these digital markers against standardized clinical instruments and outcomes in larger and more heterogeneous cohorts. Additionally, micromovement radar data could be analyzed for sleep-fragmentation detection since radar-based systems have been validated as proxies for estimating sleep-fragmentation parameters [45,46,47].

## Figures and Tables

**Table 1:** Summary of nocturia events and daytime bathroom visits for monitored users.

User	Device ID	Nocturia Events	Daytime Visits
User 1	83F8	1	49
User 2	FF2C	2	52
User 3	1100	1	24

**Table 2:** STA/LTA parameters obtained with the adaptive approach for bedroom occupancy detection in the monitored users.

User	Device ID	$T_{1}$ [s]	$T_{2}$ [s]	$\sigma_{1}$	$\sigma_{3}$	$\sigma_{2}$
User 1	EDA4	30	300	0.391	0.046	1.033
User 2	D5AC	30	300	0.252	0.049	1.139
User 3	8FFC	30	300	0.152	0.040	1.050

**Table 3:** Diagnostic comparison of bedroom-occupancy segmentation against manual STA-based annotations (07:00–22:00). $\Delta T_{\mathrm{occ}}=T_{\mathrm{occ}}-T_{\mathrm{occ}}^{\mathrm{manual}}$ (min).

User	Day	Method	$\Delta T_{\mathrm{occ}}$ (min)	$N_{\mathrm{trans}}$
User 1	13 March 2025	Adaptive	−7	12
User 1	13 March 2025	Fixed	−120	50
User 2	13 March 2025	Adaptive	−5	19
User 2	13 March 2025	Fixed	−15	21
User 3	13 March 2025	Adaptive	−5	20
User 3	13 March 2025	Fixed	−10	23

**Table 4:** Results of implementing the adaptive STA/LTA detector to obtain bedroom occupancy and detect periods of non-sedentary activity for three different users.

User	Device ID	Time in Bedroom [% of Daytime]	Non-Sedentary Time [% of Bedroom Time]
User 1	EDA4	82.8	25.0
User 2	D5AC	64.4	36.5
User 3	8FFC	50.2	34.5

**Table 5:** UWB-derived mobility metrics computed in the 8:00–22:00 window from processed trajectories using the partially constrained KF (Figure 8). Total monitored time corresponds to valid UWB data available in the window (i.e., excluding long gaps and charging periods, when applicable).

Metric	User 1	User 2	User 3
Habitual gait speed (m/s)	0.35	0.39	0.33
Maximum gait speed p95 (m/s)	0.68	1.50	0.61
Total distance (m)	61.4	80.9	60.1
Active time (hh:mm)	00:04	00:05	00:05
Sedentary time (hh:mm)	04:20	02:15	05:05
Total monitored time (hh:mm)	04:24	02:20	05:10

**Figure A1:**
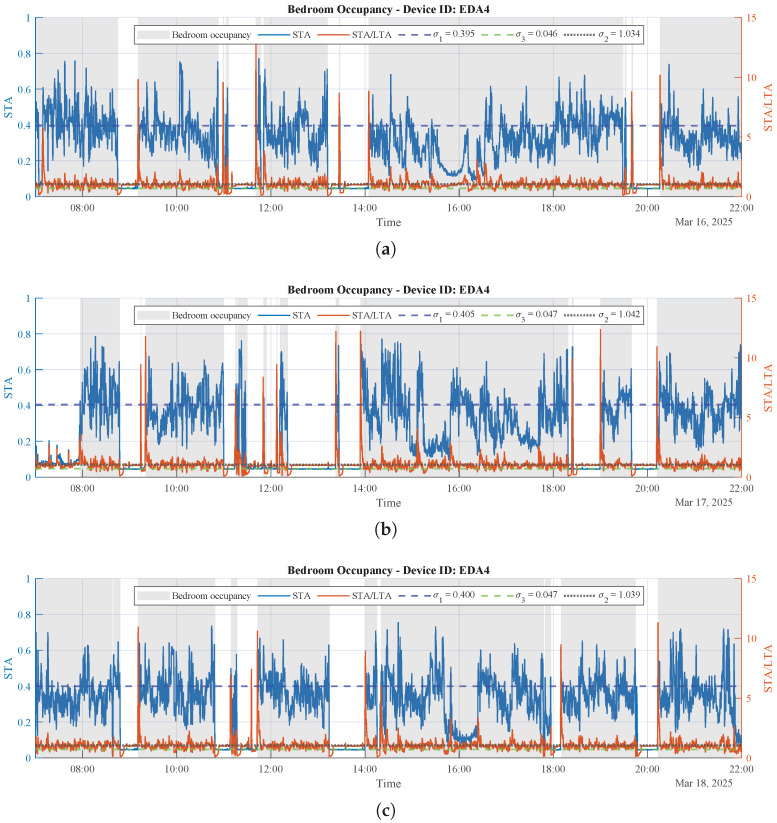

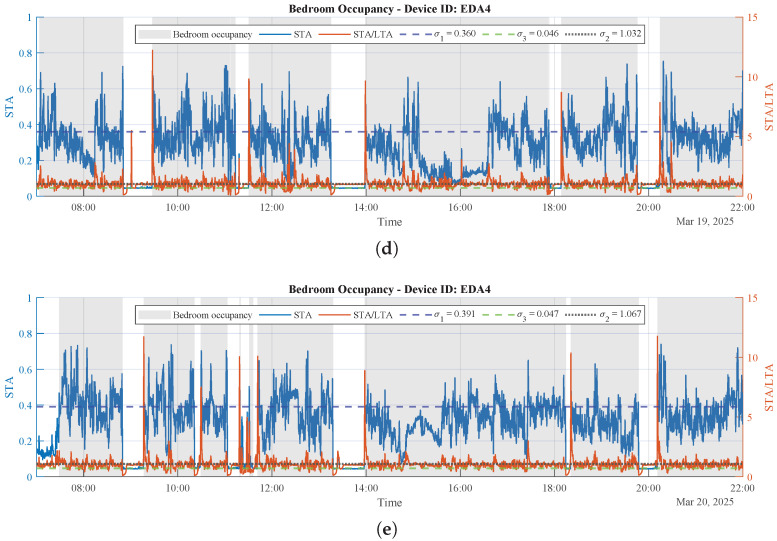
Appendix examples (User 1): adaptive STA/LTA bedroom-occupancy detection for five different days. (**a**) Bedroom occupancy for User 1—Day 1. (**b**) Bedroom occupancy for User 1—Day 2. (**c**) Bedroom occupancy for User 1—Day 3. (**d**) Bedroom occupancy for User 1—Day 4. (**e**) Bedroom occupancy for User 1—Day 5.

**Figure A2:**
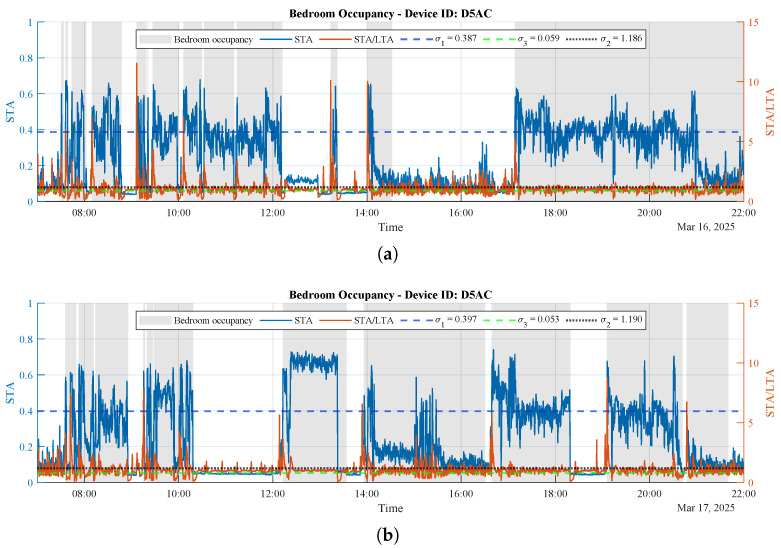

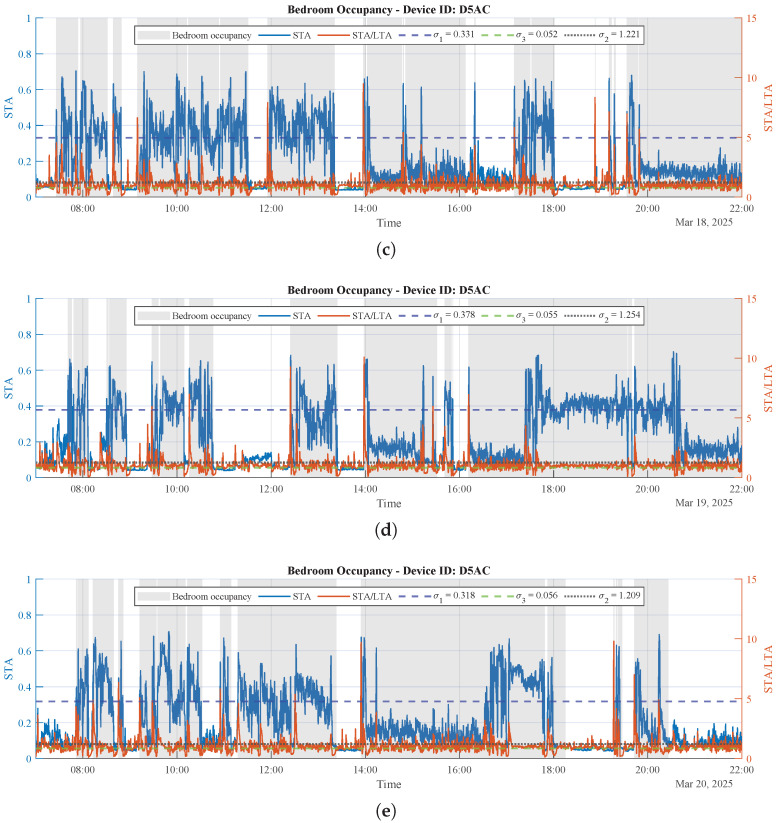
Appendix examples (User 2): adaptive STA/LTA bedroom-occupancy detection for five different days. (**a**) Bedroom occupancy for User 2—Day 1. (**b**) Bedroom occupancy for User 2—Day 2. (**c**) Bedroom occupancy for User 2—Day 3. (**d**) Bedroom occupancy for User 2—Day 4. (**e**) Bedroom occupancy for User 2—Day 5.

## Data Availability

The data presented in this study are available on request from the corresponding author.

## Abbreviations

ADL	Activities of Daily Living
BLE	Bluetooth Low Energy
CDF	Cumulative Distribution Function
CFAR	Constant False Alarm Rate
COTS	Commercial Off-The-Shelf
ECDF	Empirical Cumulative Distribution Function
EKF	Extended Kalman Filter
FMCW	Frequency-Modulated Continuous-Wave
KF	Kalman Filter
LOS	Line-Of-Sight
LPA	Light-Intensity Physical Activity
LTA	Long-Term Average
MET	Metabolic Equivalent of Task
MIA	Moderate-Intensity Activity
mmWave	Millimeter-Wave
MQTT	Message Queuing Telemetry Transport
NCV	Nearly Constant Velocity
NLOS	Non-Line-Of-Sight
OS-CFAR	Order Statistics CFAR
PANS	Positioning and Networking Stack
PIR	Passive Infrared
RF	Radio Frequency
RTLS	Real-Time Location System
STA	Short-Term Average
STA/LTA	Short-Term Average/Long-Term Average
UWB	Ultra-Wideband
VPN	Virtual Private Network

## Appendix A. Additional STA/LTA Bedroom-Occupancy Examples

This appendix presents additional qualitative examples of bedroom-occupancy segmentation produced by the proposed adaptive STA/LTA detector. Figure A1 and Figure A2 and illustrate ten representative days across two users, shown with the same visualization format as in Section 3.2.6 to facilitate direct comparison.

## References

[1] Eurostat (2024). Population Structure and Ageing—Statistics Explained. https://ec.europa.eu/eurostat/statistics-explained/index.php?title=Population_structure_and_ageing.

[2] Kim D., Bian H., Chang C.K., Dong L., Margrett J. (2022). In-Home Monitoring Technology for Aging in Place: Scoping Review. Interact. J. Med Res..

[3] Nguyen L.N., Susarla P., Mukherjee A., Cañellas M.L., Casado C.Á., Wu X., Silvén O., Jayagopi D.B., López M.B. (2024). Non-contact Multimodal Indoor Human Monitoring Systems: A Survey. Inf. Fusion.

[4] Lluva-Plaza S., Jiménez-Martín A., Gualda-Gómez D., Villadangos-Carrizo J.M., García-Domínguez J.J. (2023). Multisensory System for Long-Term Activity Monitoring to Facilitate Aging-in-Place. Sensors.

[5] García-Requejo A., Pérez-Rubio M.d.C., Villadangos J.M., Hernández Á. (2023). Activity Monitoring and Location Sensory System for People With Mild Cognitive Impairments. IEEE Sens. J..

[6] Hamäläinen M., Mucchi L., Caputo S., Biotti L., Ciani L., Marabissi D., Patrizi G. (2021). Ultra-Wideband Radar-Based Indoor Activity Monitoring for Elderly Care. Sensors.

[7] Kolakowski J., Djaja-Josko V., Kolakowski M., Broczek K. (2020). UWB/BLE Tracking System for Elderly People Monitoring. Sensors.

[8] Lluva-Plaza S., García-Domínguez J.J., Villadangos-Carrizo J.M., Jiménez-Martín A., Martínez-Becerra J. BLE-UWB Indoor Localisation for Behavioural Analysis of Community-Dwelling Older Adults. Proceedings of the 2024 IEEE International Symposium on Medical Measurements and Applications (MeMeA).

[9] Cheraghinia M., Shahid A., Luchie S., Gordebeke G.J., Caytan O., Fontaine J., Van Herbruggen B., Lemey S., De Poorter E. (2025). A Comprehensive Overview on UWB Radar: Applications, Standards, Signal Processing Techniques, Datasets, Radio Chips, Trends and Future Research Directions. IEEE Commun. Surv. Tutor..

[10] Alhazmi A.K., Alanazi M.A., Alshehry A.H., Alshahry S.M., Jaszek J., Djukic C., Brown A., Jackson K., Chodavarapu V.P. (2024). Intelligent Millimeter-Wave System for Human Activity Monitoring for Telemedicine. Sensors.

[11] Yao H., Wang X., Qi H., Liang X. Tightly Coupled Indoor Positioning Using UWB/mmWave Radar/IMU. Proceedings of the The International Archives of the Photogrammetry, Remote Sensing and Spatial Information Sciences.

[12] Fan Y., Li H., Wang H., Shi Y. (2024). An Improved Indoor Positioning Method by Fusing Ultra-Wideband and Millimeter Wave Radar Technologies. 2024 6th International Conference on Electronic Engineering and Informatics (EEI).

[13] García-Gutierrez G., Aparicio-Esteve E., Ureña J., Villadangos J.M., Jiménez-Martín A., García-Domínguez J.J. UWB and mmWave-Radar Indoor Localization System to Support Elderly Routine Analysis. Proceedings of the 2025 International Conference on Indoor Positioning and Indoor Navigation (IPIN).

[14] Labs R. (2025). RoomSense IQ Product Overview. https://www.crowdsupply.com/roomsense-labs/roomsense-iq.

[15] (2021). HLK-LD2410 24GHz Human Presence Status Sensing Module.

[16] Decawave Ltd (2017). Product Data Sheet: DWM1001C.

[17] Decawave Ltd (2017). DWM1001 System Overview and Performance.

[18] Raspberry Pi Ltd Raspberry Pi 5 Product Brief. Datasheet, 2025. Published December 2025. https://pip-assets.raspberrypi.com/categories/892-raspberry-pi-5/documents/RP-008348-DS-4-raspberry-pi-5-product-brief.pdf?disposition=inline.

[19] Pesonen J.S., Vernooij R.W.M., Cartwright R., Aoki Y., Agarwal A., Mangera A., Markland A.D., Tsui J.F., Santti H., Griebling T.L. (2020). The Impact of Nocturia on Falls and Fractures: A Systematic Review and Meta-Analysis. J. Urol..

[20] Rantz M.J., Skubic M., Koopman R.J., Phillips L., Alexander G.L., Miller S.J., Guevara R.D. (2011). Using sensor networks to detect urinary tract infections in older adults. 2011 IEEE 13th International Conference on e-Health Networking, Applications and Services (Healthcom).

[21] Kurtoğlu E., Gurbuz A.C., Malaia E.A., Griffin D., Crawford C., Gurbuz S.Z. (2022). ASL Trigger Recognition in Mixed Activity/Signing Sequences for RF Sensor-Based User Interfaces. IEEE Trans. Hum.-Mach. Syst..

[22] Gurbuz S.Z., Gurbuz A.C., Malaia E.A., Griffin D.J., Crawford C.S., Rahman M.M., Kurtoglu E., Aksu R., Macks T., Mdrafi R. (2021). American Sign Language Recognition Using RF Sensing. IEEE Sens. J..

[23] Al-Khalli N., Alateeq S., Almansour M., Alhassoun Y., Ibrahim A.B., Alshebeili S.A. (2023). Real-Time Detection of Intruders Using an Acoustic Sensor and Internet-of-Things Computing. Sensors.

[24] Kinali M., Pytharouli S., Lunn R.J., Shipton Z.K., Stillings M., Lord R., Thompson S. (2018). Detection of weak seismic signals in noisy environments from unfiltered, continuous passive seismic recordings. Bull. Seismol. Soc. Am..

[25] Tseng A. (2020). KneeArrower: Finds Cutoff Points on Knee Curves; R Package Version 1.0.0; The R Project for Statistical Computing.

[26] Antunes M., Estro T., Bhandari P., Gandhi A., Kuenning G., Liu Y., Waldspurger C., Wildani A., Zadok E. (2025). Kneeliverse: A universal knee-detection library for performance curves. SoftwareX.

[27] Sim Y., Heo J., Jung Y., Lee S., Jung Y. (2023). FPGA Implementation of Efficient CFAR Algorithm for Radar Systems. Sensors.

[28] Taillade T., Thirion-Lefevre L., Guinvarc’h R. (2020). Detecting Ephemeral Objects in SAR Time-Series Using Frozen Background-Based Change Detection. Remote Sens..

[29] Kang N., Shang Z., Du Q. (2019). Knowledge-Aided Structured Covariance Matrix Estimator Applied for Radar Sensor Signal Detection. Sensors.

[30] Zhang W., Li Y., Zheng Z., Xu L., Wang Z. (2023). Multi-Target CFAR Detection Method for HF Over-The-Horizon Radar Based on Target Sparse Constraint in Weibull Clutter Background. Remote Sens..

[31] Bull F.C., Al-Ansari S.S., Biddle S., Borodulin K., Buman M.P., Cardon G., Carty C., Chaput J.P., Chastin S., Chou R. (2020). World Health Organization 2020 guidelines on physical activity and sedentary behaviour. Br. J. Sports Med..

[32] Herrmann S.D., Willis E.A., Ainsworth B.E., Barreira T.V., Hastert M., Kracht C.L., Schuna J.M., Cai Z., Quan M., Tudor-Locke C. (2024). 2024 Adult Compendium of Physical Activities: A third update of the energy costs of human activities. J. Sport Health Sci..

[33] Schütz N., Saner H., Rudin B., Botros A., Pais B., Santschi V., Buluschek P., Gatica-Perez D., Urwyler P., Marchal-Crespo L. (2019). Validity of pervasive computing based continuous physical activity assessment in community-dwelling old and oldest-old. Sci. Rep..

[34] Mañas A., del Pozo-Cruz B., Rodríguez-Gómez I., Leal-Martín J., Losa-Reyna J., Rodríguez-Mañas L., García-García F.J., Ara I. (2019). Dose-response association between physical activity and sedentary time categories on ageing biomarkers. BMC Geriatr..

[35] Shi H., Hu F.B., Huang T., Schernhammer E.S., Willett W.C., Sun Q., Wang M. (2024). Sedentary Behaviors, Light-Intensity Physical Activity, and Healthy Aging. JAMA Netw. Open.

[36] Decawave Ltd (2016). DW1000 Metrics for Estimation of Non Line Of Sight Operating Conditions.

[37] Gualda D., Ureña J., García E. (2016). Partially Constrained Extended Kalman Filter for Navigation Including Mapping Information. IEEE Sensors J..

[38] Xiong K., Zhang T., Cui G., Wang S., Kong L. (2023). Coalition Game of Radar Network for Multitarget Tracking via Model-Based Multiagent Reinforcement Learning. IEEE Trans. Aerosp. Electron. Syst..

[39] Studenski S., Perera S., Patel K., Rosano C., Faulkner J., Inzitari M., Brach J., Chandler J., Cawthon P., Connor E.B. (2011). Gait speed and survival in older adults. JAMA.

[40] Abellan van Kan G., Rolland Y., Andrieu S., Bauer J., Beauchet O., Bonnefoy M., Cesari M., Donini L.M., Gillette-Guyonnet S., Inzitari M. (2009). Gait speed at usual pace as a predictor of adverse outcomes in community-dwelling older people. An International Academy on Nutrition and Aging (IANA) Task Force. J. Nutr. Health Aging.

[41] Soltani A., Abolhassani N., Marques-Vidal P., Aminian K., Vollenweider P., Paraschiv-Ionescu A. (2021). Real-world gait speed estimation, frailty and handgrip strength: A cohort-based study. Sci. Rep..

[42] Ikeda T., Inoue S., Konta T., Murakami M., Fujimoto S., Iseki K., Moriyama T., Yamagata K., Tsuruya K., Narita I. (2020). Can Daily Walking Alone Reduce Pneumonia-Related Mortality among Older People?. Sci. Rep..

[43] Falck R.S., Davis J.C., Liu-Ambrose T. (2017). What is the association between sedentary behaviour and cognitive function? A systematic review. Br. J. Sports Med..

[44] Lluva-Plaza S., Torres-Sospedra J., García J.J., Villadangos J.M., Jiménez-Martín A. (2023). Unsupervised Analysis of Daily Routine Evolution for Elderly People Using Room-Level Localisation. 13th International Conference on Indoor Positioning and Indoor Navigation (IPIN 2023).

[45] Toften S., Pallesen S., Hrozanova M., Moen F., Grønli J. (2020). Validation of sleep stage classification using non-contact radar technology and machine learning (Somnofy^®^). Sleep Med..

[46] Pallesen S., Grønli J., Myhre K., Moen F., Bjorvatn B., Hanssen I., Heglum H.S.A. (2018). A Pilot Study of Impulse Radio Ultra Wideband Radar Technology as a New Tool for Sleep Assessment. J. Clin. Sleep Med..

[47] Rahman T., Adams A.T., Ravichandran R.V., Zhang M., Patel S.N., Kientz J.A., Choudhury T. DoppleSleep: A Contactless Unobtrusive Sleep Sensing System Using Short-Range Doppler Radar. Proceedings of the 2015 ACM International Joint Conference on Pervasive and Ubiquitous Computing (UbiComp ’15).

